# GEIS 2013 guidelines for gastrointestinal sarcomas (GIST)

**DOI:** 10.1007/s00280-014-2547-0

**Published:** 2014-09-06

**Authors:** Andrés Poveda, Xavier García del Muro, Jose Antonio López-Guerrero, Virginia Martínez, Ignacio Romero, Claudia Valverde, Ricardo Cubedo, Javier Martín-Broto

**Affiliations:** 1 Instituto Valenciano de Oncología, Calle del Profesor Beltrán Bàguena, 8, 46009 Valencia, Spain; 2 Institut Català d’Oncologia, Avinguda de la Granvia de l’Hospitalet, 199-203, 08907 L’Hospitalet de Llobregat, Barcelona, Spain; 3 Fundación Instituto Valenciano de Oncología, C/Prof Baguena, 19, 46009 Valencia, Spain; 4 Hospital La Paz, Paseo de la Castellana, 261, 28046 Madrid, Spain; 5 Hospital Vall d’Hebrón, Passeig de la Vall d’Hebrón 119-129, 08035 Barcelona, Spain; 6 Hospital Puerta de Hierro, Calle Manuel de Falla, 1, 28222 Majadahonda, Madrid, Spain; 7 Hospital Virgen del Rocío, Avda. Manuel Siurot s/n, 41013 Sevilla, Spain

**Keywords:** GIST, Imatinib, Sunitinib, CD117, c-kit, PDFGRA

## Abstract

Gastrointestinal stromal tumors (GIST) are the most common mesenchymal soft tissue sarcoma of the gastrointestinal tract. Correct diagnosis with thorough use of pathologic and molecular tools of GIST mutations has been of the foremost importance. GIST are usually (95 %) KIT positive and harbor frequent *KIT* or platelet-derived growth factor receptor α-activating mutations. This deep molecular understanding has allowed the correct classification into risk groups with implications regarding prognosis, essential use in the development of targeted therapies and even response prediction to this drugs. Treatment has been evolving and an update to include lessons learned from recent trials in advanced disease as well as controversies in the adjuvant setting that are changing daily practice, is reviewed here. An effort from the Spanish Group for Sarcoma Research with investigators from the group has been undertaken to launch this third version of the GIST guidelines and provide a practical means for the different disciplines that treat this complex disease.

## Prologue

Gastrointestinal stromal tumors (GIST) are the most common mesenchymal tumors originating in the digestive tract. They have a characteristic morphology, are generally positive for CD117 (c-kit) and are primarily caused by activating mutations in the *KIT* or *PDGFRA* [1]. On rare occasions, they occur in extravisceral locations such as the omentum, mesentery, pelvis and retroperitoneum.

GIST have become a model of multidisciplinary work in oncology: the participation of several specialities (oncologists, pathologists, surgeons, molecular biologists, radiologists…) has allowed advances in the understanding of this tumor and the consolidation of a targeted therapy, imatinib, as the first molecular treatment that is effective in solid tumors. Following the introduction of this drug, median survival of patients with advanced stage GIST has increased from 18 to more than 60 months. Additionally, sunitinib is another targeted drug registered as second-line treatment for metastatic GIST.

## Diagnosis

### Radiology

Radiological diagnosis of GIST is similar to that of other digestive-tract tumors. In barium studies, GIST appear as submucosal lesions [2] and in ultrasound studies as hypoechogenic masses that, when large, can displace neighboring structures and show cystic, necrotic or hemorrhagic areas.

A computerized tomography (CT) scan and magnetic resonance imaging (MRI) are the first choice to study location and extension [3]. A CT with contrast and image acquisitions of the arterial and portal phases allows identification of hypervascular hepatic lesions that would otherwise go unnoticed and become evident when they turn hypodense with treatment. The latter could wrongly suggest progression due to the development of new lesions. On the contrary, a CT scan without endovenous contrast allows detection of a hemorrhage or intratumoral calcification.

In a CT, tumors appear as well-circumscribed exoluminal masses that, after the contrast, show heterogeneous enhancement, especially large tumors, which may have necrotic-hemorrhagic areas or degenerative components [4].

An MRI is useful for the local study of tumors located in the pelvic area [5] as well as for the study of the mesenteric and peritoneal extension. With PET, small GIST have a homogeneously increased uptake, while in large lesions (>4 cm), uptake may be heterogeneous [6].

### Histology

#### Techniques for histological diagnosis

The technique of choice for providing histological diagnosis is echoendoscopy-guided biopsy or a CT-guided percutaneous biopsy when the first option is not possible. Although FNA (fine-needle aspiration) endoscopy could be performed on esophagogastric tumors, this technique does not usually provide sufficient material to carry out a proper, definitive histological diagnosis, thus a biopsy would also be called for. If the biopsy become complex, a laparoscopic incision or laparotomy is required in order to obtain diagnosis. However, the use of biopsy forceps for polypectomy increases the risk of perforation and should be avoided and performed in exceptional cases only [7].

Preoperative endoscopic biopsy is not necessary when a lesion is considered suspicious, resectable or operable. On the other hand, it would be appropriate for patients with disseminated disease or in locally advanced cancers when considering neoadjuvant therapy [8].

Samples should be fixed in formaldehyde and not in Boiun’s solution as it may alter results in subsequent studies.

#### Pathological characteristics

GIST are the most common mesenchymal tumors originating in the digestive tract. They have characteristic morphological features and are generally positive for CD117 (c-kit) and have active *KIT* o *PDGFRA* mutations [1].

##### Macroscopic characteristics

*Most common sites* They are usually found in stomach (60 %), small intestine, jejunum and ileum (30 %), duodenum (5 %), rectum (2–3 %), colon (1–2 %). They are much less frequent in the esophagus (<1 %). In some cases, there is presence of disseminated tumor with unknown primary tumor and a small number of them, and the tumor originates in the omentum, mesentery and retroperitoneum [9, 10]. Metastases are typically intra-abdominal involving the peritoneum and liver; from a distance, they are odd looking and usually found on skin, bones and soft tissue.

*Morphology* The size of GIST is variable (up to 38 cm). Most measure around 5 cm at the time of diagnosis. They typically originate in the digestive tract and can be submucosal, intramural or subserosal. They are rarely invasive and there is often ulceration of the mucous membrane with poor prognosis [10]. Necrotic, hemorrhagic and cystic degeneration areas are usually displayed [11]. They are usually solitary, sporadic cases usually have multiple lesions [12] in familial or neurofibromatosis GIST and Carney Triad [13]. Its growth pattern is extensive (21 %) and pseudo-extensive (45 %) or infiltrative (24 %). The pathology report must always include three-dimensional tumor measurement, and the existence of quantification of necrosis and distance between lesion and margin as incomplete resection is associated with poor prognosis [14].

##### Microscopic characteristics

Three histological types can be distinguished according to the cellular appearance: *fusiform* cells (77 %), *epithelioid* cells (8 %) and *mixed* (15 %) (1). The epithelial type is more frequently observed in stomach and epiplon [15].

The number of mitosis can vary substantially, between 0 and over 150 mitoses per 50 high-power fields (hpf). Most show a low or very low mitotic index (≤5 mitosis/50 hpf). The method for counting mitoses in the most active areas, in a total of 50 hpf (corresponding to an area 10 mm^2^), should be standardized given its prognostic relevance. Strict criteria should be followed as pyknosis and karyorrhexis must not be overlooked. The mitotic index should be graded as follows: *low* ≤5/50hpf and *high* >5/50hpf [16].

##### Immunohistochemistry

Over 95 % of GSIT have CD117 (c-kit) expression with diffuse cytoplasmic staining pattern but also rarely in the membrane or Golgi apparatus. There is intense staining in 75 % of cases. Moreover, 70–90 % also express CD34, 20–30 % actin, 8–10 % S-100 and desmin in 2–4 % [1]. IHC studies are useful in confirming a diagnosis of GIST and given the implications of diagnosis, appropriate CD117 immunohistochemistry in order to avoid errors. Staining of Ki67 is a prognostic factor and recommended [17]. DOG1 can optionally be included in the initial panel and is highly recommended in negative c-kit [18], in which DOG1 is expressed in over 35 % of cases.

##### Differential diagnosis

The main differential diagnosis in fusiform GIST comprises smooth muscle tumors (leiomyoma and leiomyosarcoma); schwannoma and malignant peripheral nerve sheath tumor; inflammatory myofibroblastic tumor; solitary fibrous tumor, sarcomatoid carcinoma; inflammatory fibroid polyp and desmoid fibromatosis. Differential epithelioid GIST diagnosis includes poorly differentiated carcinomas; endocrine cancers and variants of epithelioid leiomyosarcoma and malignant peripheral nerve sheath tumor. Luckily, morphological features together with an adequate immunohistochemical panel allow proper diagnosis [11].

##### Kit-negative GIST

Between 4 and 5 % of GIST with typical morphological features are negative for CD117 [19, 20]. Those with negative stains or weak staining <10 % of tumor extension are to be considered as such. Kit-negative GIST is clinically, pathologically and genetically different from kit-positive GIST. Although they are more frequently found in stomach, they can also be observed in the omentum and peritoneal surface. They are less commonly CD34 and actin positive, while desmin expression is approximately 30 %, especially in stomach lesions and epithelioid morphology [18]. DOG1 positive was observed in slightly over one-third of tumors [18]. Other antibodies such as PCK tetha or PDGFRA have been found not to be very reproducible or useful [21, 22].

Kit-negative GIST present a true diagnostic challenge. It is recommended to extend the immunohistochemical panel with other markers such as DOG1 and a mandatory study for *KIT* and *PDGFRA* mutation, being mindful that there is a small percentage of GIST with typical morphology, negative for CD117 and DOG1 and wild type for *KIT* and *PDGFRA* genotype [20]. Cases morphologically defined as atypical, cellular atypia, CD117 and DOG1 negative and with no *KIT* or *PDGFRA* mutations should not be classified as GIST.

#### Final recommendations

- Pathologic diagnosis is based on both unique microscopic features and ancillary techniques (CD-117, CD34, actin, desmin, S-100 and ki-67), which are very important to confirm diagnosis.
- The pathology report must include tumor size; number of mitoses per 50 HPF (10 mm^2^) counted in the most active regions; and margins status.
- It is advisable to refer the complex or unusual cases to experienced centers.
- Regarding tumors with typical morphology GIST, an extended phenotype of DOG1 as well as *KIT* and *PDGFRA* gene mutation analysis is required.
- Albeit optional, it is convenient to include the risk group separated by site. Table 1 (Miettinen et al.) [23] and histologic grading defined exclusively by the number of mitosis (low grade ≤5/50HPF, high grade >5/50HPF).

**Table 1:** Primary gastrointestinal stromal tumors (GIST) risk assessment guidelines

Tumor parameters		Risk of progression* (%)	
Mitotic index	Size	Stomach***	Small bowel***
≤5 per 50 high-power field (HPF)	≤2 cm	No (0 %)	No (0 %)
	>2–≤5 cm	Very low (1.9 %)	Low (4.3 %)
	>5–≤10 cm	Low (3.6 %)	Moderate (24 %)
	>10 cm	Moderate (10 %)	High (52 %)
>5 per 50 HPF	≤2 cm	No^**^	High^**^
	>2–≤ 5 cm	Moderate (16 %)	High (73 %)
	>5–≤10 cm	High (55 %)	High (85 %)
	>10 cm	High (86 %)	High (90 %)

^*^ Defined as metastasis or cancer-related death

^**^ Small number of cases

^***^ See stomach for omentum and other locations (esophagus, colon, peritoneum and mesentery) see small bowel

### Molecular diagnosis

GIST are characterized by activating mutations in *KIT and PDGFRA* genes—are shown to be mutually exclusive encoding a receptor tyrosine kinases type III (RTC) [11, 12]. *KIT* mutations are found in 60–85 % of GIST tumors, while *PDGFRA* mutations are found in 5–10 %. Approximately 10–15 % of GIST do not have detectable mutations in any of these receptors (GIST wild type), suggesting that other molecular routes can also be involved in the pathogenesis of these tumors [24–26].

#### Spectrum of mutations in GIST

Mutations found in GIST mainly affect exons which codify functional domains of KIT and PDGFRA receptors. Among the main types of mutation, we find the following: deletions, point mutations, duplications, insertions and complex mutations [19].

Mutation detection before tyrosine kinase (TK) inhibitor therapy such as imatinib is known as primary mutation (and mainly affects exons 11, 9, 13 and 17 of *KIT*, and exons 18, 12 and rarely affects 14 of *PDGFRA*). Meanwhile, mutations detected during treatment, which are to a large degree responsible for resistance to TK inhibitors, are known as secondary mutations (generally detected in exons 13, 14 and 17 of KIT and 18 of PDGFRA) [24, 26].

##### KIT mutations

The most common *KIT* mutations affect exon 11 (juxtamembrane domain). Approximately 70 % of GIST present some type of mutation in this exon [24, 27]. The most frequent mutations in this exon are interstitial deletions, commonly affecting the beginning of exon 11 (between codons 550 and 579) and especially codons 557–559. Then, there are point mutations, albeit with a lower incidence, and limited to four codons (557, 559, 560 and 576). Lastly, at the extreme end of the exon (between codons 571 and 591) and in a much smaller proportion of patients, we find tandem duplications associated with GIST gastric site and epithelioid or mixed cell morphology [25, 28–30].

In exon 9 (*extracellular domain*), only duplication of residues 502–503 have been described and is present in 9–20 % of cases depending on the study. This mutation is mainly associated with GIST of small bowel location and greater malignant potential [26, 30].

The *KIT*-*TK domains* are encoded by exons 13 and 17. Only point mutations have been found in these exons, the frequency being between 0.8 and 4.1 % for exon 13 lower than 1 % in the case of exon 17 [25, 26, 30–32].

##### PDGFRA mutations

Overall, the estimated frequency rate of *PDGFRA* mutations in GIST is 5–10 % [24, 25, 33], which are associated with localized gastric GIST and epithelioid morphology [25, 27, 33]. Mutations are concentrated in the *juxtamembrane domain* (0.7 %*)* encoded by exon 12; in *TK domain* (6 %) encoded by exon 18, DD842 V mutation being the most frequent (65–75 %); and very rarely in exon 14 (0.1 %) [24, 25, 27, 33].

##### GIST wild type

Around 12–15 % of adult GIST and 90 % of pediatric GIST lack *KIT* and *PDGFRA* mutations [19]. Molecular pathogenesis and tumor biology of this subgroup represent one of the greatest areas of speculation and investigation in which the involvement of other TK receptors, such as IG1R, has already been demonstrated [34]. Besides, other intracellular signaling pathways as the one controlled by BRAF, with mutations described in 7 % of wild-type GIST [35] and mutations in the succinate dehydrogenase enzymatic complex subunit genes (SDHC), are mostly associated with germline mutations [36].

#### Kit-negative GIST

Approximately 5 % of GIST are c-kit negative, leading to diagnostic difficulties. Between 30 and 50 % of these tumors present mutations in *KIT* or *PDGFRA [* 27, 37–39], which may have therapeutic implications. The notion that a GIST can be negative for c-kit as well as wild type for *KIT* and *PDGFRA* mutations is not entirely clear considering that current diagnosis is performed by exclusion [27]. Furthermore, the last European consensus proposed, using a mutational analysis of *KIT* and *PDGFRA,* to confirm GIST diagnosis, especially in CD117/DOG1 negative cases [40].

#### Syndromes associated with GIST

At present, there are many syndromes associated with GIST, among them:

1. Carney Triad: characterized by gastric GIST, paraganglioma, pulmonary chondroma, which may develop in any age group, making it difficult to discard this condition in pediatric *wild*-*type* GIST [41].
2. *Neurofibromatosis Type* *1*: usually marked by a wild-type GIST predominantly located in the small intestine and quite possibly a multicenter study [42].
3. *Carney*–*Stratakis syndrome:* characterized by germline mutations in some subunits of the succinate dehydrogenase enzyme (SDHB, SDHC y SDHD) producing a dyad of GIST and paraganglioma [43, 44].

#### Final recommendations

The last multidisciplinary ESMO consensus [40] recommends including a molecular systematic analysis in the diagnosis of all GIST (specially advanced GIST), given the type of relevant predictive and prognostic information provided and required in cases of GIST without CD117 and DOG1 expression. In these cases, it is recommended to refer patients to a reference center with their own laboratory, integrated in quality assurance programs and with proven experience.

## Predictive factors in locally advanced or metastatic disease

### Genotype correlation in primary disease with therapeutic results with imatinib for first line

Patients with an exon 11 KIT mutation have a better chance of responding, a longer time to progression (TTP) overall survival (OS) versus those with exon 9 mutations or *wild* *type* [45–47]. Moreover, the meta-analysis carried out in 2 phase II trials (EU-AUS and US-CDN) comparing 400 versus 800 mg of daily imatinib in patients with metastatic or non-resectable GIST, showed that patients with exon 11 mutation had a better progression-free survival (PFS) and overall survival with respect to exon 9 mutation or wild type [41].

### Genotype correlation in primary disease with therapeutic results imatinib dosage

In the above-mentioned meta-analysis, it was found that mutations in exon 9 were the only predictive factor for imatinib response with greater benefit, statistically significant for PFS patients who received high doses of imatinib. A 31 % lower risk of death was observed in favor of 800 mg, although it did not reach statistical difference [48]. These findings confirm data previously disclosed by the European study. Unlike the American study, which only had 32 patients with exon 9 mutations, the European one showed 58 patients with same mutations [48, 49].

### Primary genotype correlation with therapeutic results with sunitinib as second-line therapy

Sunitinib inhibits multiple receptor tyrosine kinases (VEGFR, PDGFR, KIT, FLT3) and has a higher receptor binding affinity than imatinib. Data obtained from Phase I/II clinical trials of 78 patients (out of 97) treated with sunitinib as second line and with preimatinib genotype information showed that patients with exon 9 mutations and wild-type tumor were associated with a more favorable outcome (partial response and disease stabilization over 6 months) compared with those with exon 11 mutations. Furthermore, the median PFS was significantly higher in patients with primary exon 9 mutation (19.4 months; *p* = 0.0005) or w*ild type* (19 months; *p* = 0.0356) regarding those with exon 11 mutations (5.1 months). A significantly greater OS was obtained, comparing those with exon 9 mutations, (26.9 months; *p* = 0.012 and 30.5 months; *p* = 0.0132) for *wild type*, as opposed to those with exon 11 mutations (12.3 months) [49].

### Secondary genotype correlation with therapeutic results for sunitinib

It is acknowledged that secondary resistance observed in patients with metastatic GIST is mostly developed due to the appearance of secondary mutations. Interestingly, no secondary mutations were found in initially wild type and resistant patients [50].

In vivo data obtained from biopsies carried out at the time of progression to imatinib provide important information on secondary mutations identified in 64 % of the available cases. Significant clinical benefit as well as RFS was better in cases with secondary mutations localized in exon 13 or 14 with regard to those in exon 17 or 18 [12].

### Mechanisms of resistance to imatinib

Resistance to imatinib is a major therapeutic problem as, among patients who fail to respond to initial imatinib (primary resistance, 5–15 %) or stop responding (secondary resistance), barely 5 % respond to conventional treatments. Primary resistance can be defined as that occurring in the first months of imatinib therapy. Progression is typically multifocal, and mutations are frequently found in exons 9 and WT. The mechanisms for secondary resistance to imatinib are heterogeneous and can be grouped into various groups [51, 52]:

- Acquisition of secondary KIT mutations
- *KIT* gene amplification
- Activation of alternative signaling pathways
- Functional resistance due to *KIT* or *PDGFRA* activation in absence of secondary mutations.

The most common mechanism is the appearance of a new mutation. Secondary mutations in exons 13, 14, 17 or 18 account for 62 % of GIST with primary in KIT exon 11 mutations but only in 16 % have a primary mutation of exon 9. Moreover, no secondary mutations appear in GIST which do not have a primary mutation in *KIT* or *PDGFRA* [49]. There is evidence of clonal and/or polyclonal evolution of secondary mutations in a small proportion of patients (18.8 %). In this way over time, the same patient may develop secondary mutations in different tumor resistant implants. In view of this finding, the therapeutic approach of these patients will have to be taken into account [50, 53].

### Final recommendations

The clinical application of secondary mutation findings is not so clear-cut at the present time and should be subject to further research studies.

Lastly, given the interest in translational studies regarding this neoplasm, it is advised to take fresh tissue for the application of new technologies of molecular pathology, which will ultimately have a positive impact on the patient.

## Treatment

It is highly advised that diagnostic and therapeutic processes could be referred to expert teams for GIST care [54].

### Localized disease

#### Surgery for localized disease

Complete surgical resection is the standard treatment for localized GIST. Radiological criteria for unresectability include infiltration of the celiac trunk, the superior mesenteric artery or mesenteric artery-to-portal vein. Lymphadenectomy is unnecessary given the low frequency of lymph node metástasis or metastasis. Some exceptions could be SDH-deficient GIST especially in pediatric population.

The aim was to achieve a R0-type surgery (optimal surgery), complete removal leaving an intact capsule. It is therefore necessary, in some cases, to remove neighboring organs and perform a surgical “block excision.” Segmental resection of intestine and stomach is accepted, and thus, aggressive and a more extensive surgery to remove unaffected tissue is unnecessary [55].

Regarding R1 resection (marginal excision containing tumor cells), reexcision could be offered and shared with the patient, if this does not imply major functional squeals. If the context of R1 surgery is a very low- to low-risk tumor, the physician should communicate the wait-and-see approach to the patient as opposed to aggressive surgery with permanent damage since there is no clear evidence that R1 margins entail a worse prognostic in such cases.

Peritoneal and hepatic surfaces should be carefully examined during a laparotomy to rule out tumor spread. Tumor resection must be carefully performed to avoid tumor rupture. In this regard, a laparoscopic approach is strongly discouraged in patients with voluminous tumors.

#### Prognostic factors after surgery in localized GIST

**Fig. 1:**
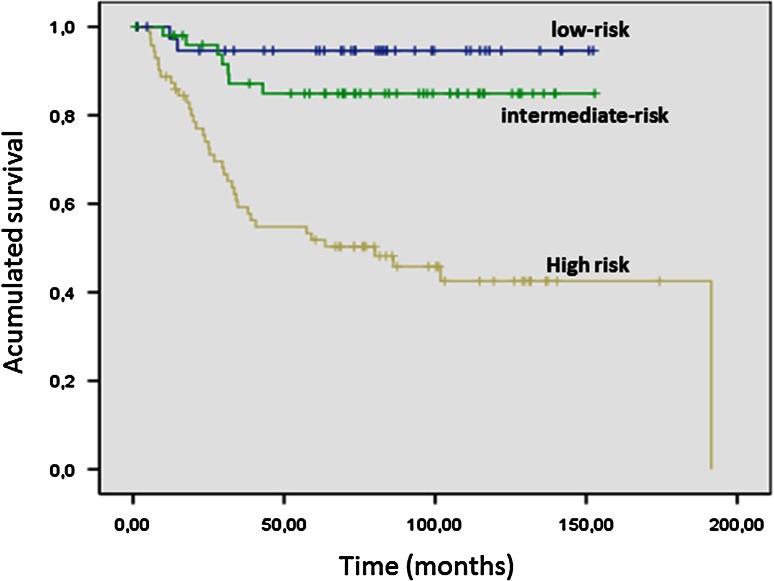
Relapse-free survival analysis according to Fletcher’s risk groups in 162 patients registered in GEIS

Relapse-risk assessment for primary GIST is paramount not only providing prognostic information when trying to determine risk factors but also estimating the potential benefit of adjuvant imatinib. In 2002, an index was proposed (NIH Consensus NIH or Fletcher) [15] based on studies of prognostic factors studies for patients with localized GIST, to estimate the risk of recurrence (Table 2; Fig. 1), based on the number of mitosis per 50 high-power fields (HPF), the size of the primary tumor and the two variables with the greatest prognostic significance. Principally, it seems that any GIST has malignant potential and the index makes it possible to classify GIST patients according to risk factors and complete resection.

**Table 2:** Risk groups for gastrointestinal stromal tumors according to Fletcher et al

	Size^*^	Mitotic index (50 HPF)**
Very low-risk	<2 cm	≤5 mitosis
Low-risk	2–5 cm	≤5 mitosis
Intermediate-risk	≤5 cm	6–10 mitosis
	5–10 cm	5 mitosis
High-risk	>5 cm	>5 mitosis
	>10 cm	Any number of mitosis
	Any size	>10 mitosis

^*^Size takes into account the maximum dimension. Variation is accepted with the measurement of tumors before or after fixation and the existing differences among observers

^**^50 HPF represent between 10 and 12 mm^2^ in current optical density. Ideally, the mitotic index should be expressed according to the surface to be examined based on the power field magnification (HPF)

Subsequently, Miettinen et al., analyzed data of 1.765 patients with gastric GIST and observed that patients only developed metastasis in 2–3 % tumors with <10 cm and <5 mitosis/50 HPF, compared with 68 % of those who presented >10 cm and >5 mitosis/50 HPF [10]. A second series including 906 patients with <10 cm and <5 mitosis/50 HPF tumor located in the jejunum and ileum, presented recurrence in 24 % compared to 90 % which presented >10 cm and >5 mitosis/50 HPF tumor.

**Fig. 2:**
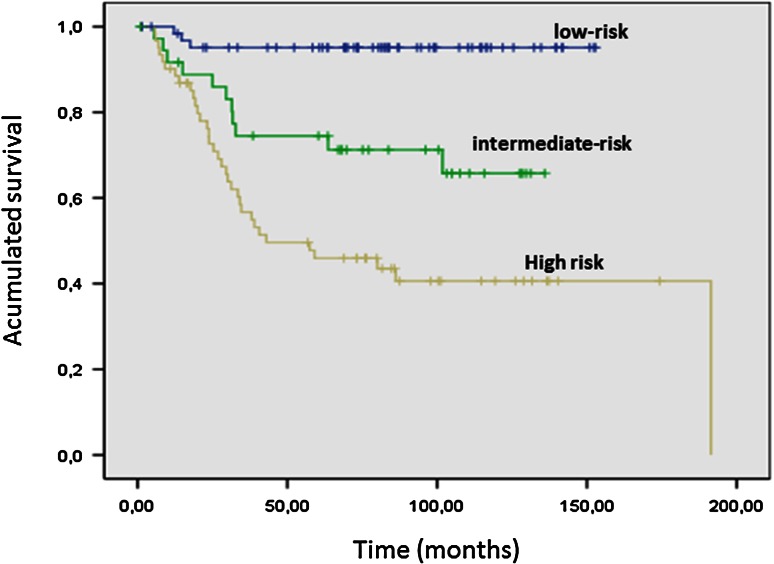
Disease-free survival analysis according to Fletcher’s risk groups in 162 patients registered in GEIS

Based on this data, these same authors put forward a new risk index (AFIP/Miettinen) that includes anatomic site [23]. This classification better reflects the high-risk population than the Fletcher index (Table 3; Fig. 2), especially between the intermediate and low-risk groups. The risk of gastric cancer relapse varies from 2 % in tumors with <5 mitosis per 50 HPF to 90 % in gastrointestinal tract GIST with tumors more than <10 cm and <5 mitosis/50 HPF. The casuistry of GEIS group has shown that the Miettinen’s classification exhibited statistical significance for discriminating low, intermediate and high-risk groups. This was not the case when the Fletcher classification was used [29].

**Table 3:** Risk groups for gastrointestinal tumors adapted by Miettinen et al

	Size	Mitotic index (50 HPF)^*^	Location
Very low risk	2–5 cm	≤5 mitosis	Gastric
Low risk	>5 years ≤10 cm	≤5 mitosis	Gastric
	2–5 cm	≤5 mitosis	Intestinal
Intermediate risk	>10 cm	≤5 mitosis	Gastric
	>5 years ≤10 cm	≤5 mitosis	Intestinal
	2–5 cm	>5 mitosis	Gastric
High-risk intestinal	2–5 cm	>5 mitosis	Intestinal
	>10 cm	≤5 mitosis	Intestinal
	>5 years ≤10 cm	>5 mitosis	Gastric
	> 10 cm	>5 mitosis	Gastric
	>5 years ≤10 cm	>5 mitosis	Intestinal
	>10 cm	>5 mitosis	Intestinal

^*^50 HPF represents an area of 5 mm^2^ in the optical fields used by Miettinen

The main differences between both classification systems lies in patients with gastric GIST, larger than 10 cm but with <5 mitosis per HPF. Using Fletcher’s classification, the latter would be in the high-risk group with a recurrence-free survival (RFS) of 50 % at 5 years. Nevertheless, they would fall within the intermediate-risk category with a RFS of 80 % according to the Miettinen group classification.

On the other end of the spectrum we find GIST tumors with extragastric location of <5 cm and more than 5 mitosis per HPF. According to Fletcher’s classification, they would fall within the intermediate group with a RFS probability of 85 % versus being in the high-risk group with 45 % RFS in the Miettinen group classification. It is important to note that Miettinen considered a total area 5 mm^2^ in 50 fields HPF characterized by the use of different optical components, while in practice 50 HPF typically corresponds to a total area of 10 mm^2^. Therefore, if we use Miettinen’s risk classification, we should also make the correction of dividing the number of mitosis by half including the current optical elements by 50 HPF.

Other succeeding risk classifications such as the American Joint Committee on Cancer (TNM) [56] or the nomogram [57] for the individual risk assessment show some differences such as the anecdotal evidence of ganglionic extension or the selection bias that encumber studies in some large centers and magnify the likelihood of relapse.

Joensuu H. recently introduced a capsule rupture classification known as modified NIH that simplifies the site classification (gastric/non-gastric) but at the same time renders heat maps to be more complex as categorization of continuous variables is not used [58].

The NCCN [59] and ESMO [40] guidelines tend to favor Miettinen’s classification when capsular rupture is considered comparable to peritoneal dissemination.

A further problem posed, at least theoretically, is regarding adjuvant imatinib clinical trials designed using Fletcher’s risk classification. If we were to adopt a more liberalized stance on drugs, we would recommend adjuvant imatinib treatment for patients with gastric GIST for tumors of 10 cm or larger with <5 mitosis/HPF (considered as high risk according to Fletcher), when the risk of recurrence is 65 %. Therefore, the most rational approach should bear in mind the most current prognostic information in which the high risk of recurrence category is more accurate.

Although GIST tumors are a model for the so-called molecular target therapies, molecular prognostic factors have not been incorporated in the risk of recurrence classifications. There is available evidence indicating that the type and location of the mutation has an effect on the risk of recurrence. Deletions affecting exon 11, codon 557/558 of the c-KIT gene, have a higher recurrence risk and it will occur within the first 3–4 years after surgery [29, 60]. The leading role of “critical mutation” has been confirmed in recent series [58, 61].

#### Final recommendations

- We recommend the use of the risk group classification proposed by Miettinen as it is the best at identifying low, intermediate and high-risk populations. Spontaneous or intraoperative capsule rupture should be considered as a very poor prognostic factor.
- Deletion type of mutations affecting codons 557 and 558 confers a risk for recurrence regardless of its previous classification, according to our experience. The risk is greatest within the first 30 months after surgery and then drops drastically. Nevertheless, results from other prospective studies are needed in order to assess the value of this variable.

### Adjuvant treatment

#### Imatinib trials overview

Despite the fact that complete resection is feasible in most localized GIST cases, there is still a recurrence rate of up to 50 % according to some series. The role of imatinib as adjuvant treatment to prevent recurrence has therefore been assessed in several clinical trials. Evidence derived from the large Phase III randomized trials ACOSOG Z9001 [62] and SSGX-VIII/AIO [63], has shown a relapse-free survival (RFS) benefit with imatinib. Moreover, the SSGX VIII/AIO study showed an increase in overall survival (OS) with 3 years of imatinib administration over 1 year in high-risk patients (in accordance with NIH modifications). Preliminary results of the first interim analysis of EORTC 62024/GEIS-10 study [64] have recently been communicated in ASCO 2013. This phase III trial included intermediate and high-risk patients randomized at 2 years with imatinib over observation.

Although the initial end point was OS, it was changed to time to imatinib failure (TIF) in 2009 due to the small number of relapses in the control group. No significant statistical differences were found in either arms (OS and TFI) after a 4.7-year follow-up. Nevertheless, there was an objective tendency to an improved TFI in high-risk patients (in both NIH 2002 as well as modified NIH classifications). A benefit was also observed in RFS as previous reported studies in favor of adjuvant treatment with imatinib in high-risk patients.

In view of these results, both NCCN and ESMO guidelines as well as consensus of the scientific community, recommend 3 years of adjuvant treatment with imatinib in high-risk patients. Adjuvant treatment for low-risk patients is not indicated. However, currently there is not enough scientific evidence to support adjuvant treatment with imatinib in intermediate-risk patients. Based on these considerations, for uncertain cases, it is important to carry out an assessment of risk of recurrence and properly classify them by using modified classification tools (modified Miettinen classification of Joensuu H).

There are still many unclear areas concerning duration of adjuvant treatment and whether more than 3 years of treatment would increase benefit in patients at higher risk. Studies like PERSIST-5 may shed some light on this issue. Moreover, another aspect that needs to be clarified is whether relapse is actually avoided or just delayed, given the relapses observed in SSGX-VIII/AIO following adjuvant treatment interruption at 6–12 months in both arms [63].

#### Special cases

- Capsule break: These are generally accepted as disseminated patients given that 100 % will relapse, at least on a peritoneal level. Therefore, imatinib administration is recommended as advanced disease setting.
- Specific genotypes: Adjuvant imatinib is not recommended in patients with D842 V PDGFRα mutation given its known resistance to it. There is no consensus regarding the benefit of a daily dose of 400 mg of imatinib for carriers of an exon 9 mutation in the KIT gene. The efficacy of a daily dose of 800 mg of imatinib was extrapolated from the evidence of disseminated disease. Nonetheless, this scenario has not been proven in clinical trials and therefore has not been approved for adjuvant treatment. Survival in patients with wild type does not seem to increase with the use of adjuvant imatinib, and thus, there is still controversy over imatinib administration and each case must be considered individually.
- Patients with R1 surgery: There is no evidence confirming the benefit of adjuvant imatinib in low-risk patients with affected microscopic margins. Surgical reexcision could be considered for these cases (see surgical section).

#### Final recommendations

- High-risk patients: 3 years of adjuvant treatment with imatinib is recommended.
- Low-risk patients: adjuvant treatment is not indicated.
- Intermediate-risk patients: currently, there is not enough scientific evidence to support adjuvant treatment with imatinib. For uncertain cases, it is important to carry out an assessment of risk of recurrence and properly classify them by using modified classification tools (modified Miettinen classification of Joensuu H).

### Advanced disease

#### Treatment of unresectable or metastatic disease

##### Dose and efficacy of imatinib treatment

Gastrointestinal stromal tumors have been a paradigmatic example of chemo-resistant tumors with <5 % of responses and 14 months as the median of survival reported in the literature. Imatinib mesylate (STI571, Gleevec™, Novartis Pharmaceuticals, Basel, Switzerland) is a selective tyrosine kinase inhibitor (TKI), whose targets include ABL, BCR-ABL, KIT and PDGFR and constitutes as a very effective agent for the treatment of clinically advanced, metastatic or surgically unresectable GIST [65, 66].

The standard dose of imatinib of 400 mg/m^2^ per day was established from two different randomized phase III trials in metastatic GIST with positive immunostaining for kit (EORTC-ISG-AGITG y NASG-S0033). In both trials, daily doses of 400 versus 800 mg were compared without any survival difference and with a more favorable toxicity profile for lower doses. The clinical benefit rates (CR, PR and SD) for 400 and 800 mg were 90 and 88 %, respectively, in NASG-S0033 study. These rates were 91 and 87 %, respectively, in EORTC-ISG-AGITG study. Furthermore, there was statistically significant difference, in terms of progression-free survival (PFS), favoring 800 mg dose in an European trial: progression-free rate at 2 years 52 versus 44 % (HR 0.78) [67, 68]. In a meta-analysis, analyzing 1,640 patients enrolled in the mentioned trials, a slight but still significantly advantage was found in terms of PFS for the high-dose arm [48]. Nevertheless, no survival advantage was detected and thus the standard dose, as for general recommendation, is 400 mg daily.

##### Predictive value of genotype for imatinib efficacy

Interestingly, one of the notable features of the clinical studies of imatinib for GIST treatment is the consistent observation that defined subsets of GIST according to their mutational status have different outcomes during treatment and therefore should be considered in devising treatment strategies.

Responses to imatinib depend on the functional domain affected [69]. Table 4 lists the correlation between tumor genotype and objective response (both complete and partial responses) in four trials (phase I–III). On the basis of 768 genotyped GIST, the objective response rates for KIT exon 11, exon 9 mutants and GIST WT are 72, 38 and 28 %, respectively [46, 70, 71]. Likewise, the probabilities of primary resistance to imatinib for KIT exon 11, KIT exon 9 and WT GIST are 5, 16 and 23 %, respectively (Table 4). An even more striking observation is that KIT and PDGFRA mutational status correlates with time to progression (TTP) and overall survival (OS), with superior survival seen for patients with GIST carrying an exon 11 KIT mutation. For example, in the American phase III trial, the median TTP for patients with GIST harboring KIT exon 11, KIT exon 9 and WT was 25, 17 and 12, 8 months, respectively. A similar OS benefit was seen for patients with KIT exon 11 mutations (60 months) compared with those observed for KIT exon 9 (38 months) or WT (49 months) genotypes. Comparable results regarding TTP, OS and KIT mutational status were also observed in the European/Australasian phase III trial [46].

**Table 4:** Relationship between *KIT* mutational status, response rate and outcome on imatinib therapy

		European phase I/II (*n* = 37)	B2222 phase II (*n* = 127)	European/Australian Asian phase III (*n* = 363)	North American SWOG S0033 phase III (*n* = 324)	Weighted average
		% (*n*)	% (*n*)	% (*n*)	% (*n*)	% (*n*)
*Objective response* ^a^						
*KIT* exon 11		83 (24)	83^b^ (85)	70^b^ (248)	67^b^ (211)	71 (568)
*KIT* exon 9		25 (4)	48 (23)	35 (58)	40 (25)	38 (110)
No mutation		33 (6)	0 (9)	25 (52)	39 (33)	28 (100)
*Progressive disease*						
*KIT* exon 11		4 %	5 %	3 %	8 %	5 %
*KIT* exon 9		0 %	17 %	17 %	16 %	16 %
No mutation		33 %	56 %	19 %	21 %	23 %

^a^Defined as complete or partial response by SWOG (B2222) or RECIST criteria (all other trials); excluded non-evaluable patients

^b^Statistical difference between *KIT* exon 9 and no mutation group

On the other hand, the meta-analysis also confirmed the observations previously reported in the European/Australasian trial, and therefore, it was concluded that KIT exon 9 mutations constituted a dose-dependent predictive factor for imatinib treatment identifying patients with a better response to high doses of imatinib (400 mg twice daily). Consequently, the estimated risk of progression for patients with KIT exon 9 mutations was drastically reduced (42 %; *p* = 0.0017) in the 800-mg/day arm compared with the 400-mg/day dose of imatinib. In the same direction, the risk of death was also reduced in a 31 % in this subgroup of patients.

Only small numbers of patients with GIST harboring *PDGFRA* mutations were included in the original phase I–III trials. On the basis of in vitro data, the most common *PDGFRA* mutation in GIST, D842 V, is fully resistant to the effects of imatinib [33]. Among the patients whose GIST harbored a *PDGFRA* D842 V mutation in the American phase III trial, there were no objective responses and stable disease was observed for a few months in some of the patients. From in vitro experiments, dasatinib showed activity in GIST cell lines with this specific mutation [72], somewhat recently confirmed in the clinical setting [73].

Thus, taking together the previous information, there is a consensus in the following recommendations:

- Genotype is mandatory for treating advanced/metastatic GIST patients. Evidence II, A.
- Imatinib 400 mg/day is the recommended dose in first line in advanced/metastatic GIST. Evidence I, A.
- In exon 9 mutants, imatinib 800 mg/day is the recommended dose. Evidence III, A.
- In *PDGFRA/KIT* WT GIST is not clear enough that imatinib should be the standard.
- In imatinib-resistant D842 V mutant, alternative treatments other than imatinib could be advised (i.e., dasatinib). Evidence IV, B.

##### Practical issues on imatinib as first line in GIST

1. How long should the therapy last? The BFR14 trial, which randomized patients with nonprogressive GIST to continuation versus interruption of imatinib after 1, 3, or 5 years of treatment, showed that treatment interruption was associated with a high risk of progression even in patients with a complete response [13]. Interestingly, although imatinib rechallenge could control the disease in most patients, the quality of the tumor response rarely reached that before treatment interruption. Consequently, in patients with metastatic or unresectable GIST, imatinib should be continued until disease progression even when metastatic lesions have been previously surgically excised or until unacceptable toxicity. Evidence II, B.
2. Compliance. Although imatinib is usually a well-tolerated drug with as few as 2 % of grade III–IV adverse events, the long duration of therapy and persistent grade I–II side effects could impact in treatment compliance and consequently in disease outcome. Therefore, a good education of patients regarding the importance of compliance and potential interactions with other drugs or foods as well a proper and prompt management of side effects is crucial.
3. Imatinib plasma levels. Although it remains to be demonstrated in a prospective setting, retrospective data suggest that low plasma levels at steady state are associated with a worse outcome. So, the median time to progression was 11.3 months for patients with imatinib plasma levels <1, 11 ng/mL compared to more than 30 months for patients with plasma levels above that threshold [14]. Plasma levels could be especially useful in case of suspected bad compliance as the cause of tumor progression, in patients at risk of potentially important interactions with other concomitant drugs or unexpected toxicities. Evidence IV, B.
4. Rechallenge of imatinib after adjuvant treatment. For patients recurring during adjuvant treatment, second-line treatments including imatinib 800 mg/day should be discussed. For those patients relapsing with metastatic or unresectable disease after imatinib interruption, although no direct prospective evidence is available, based on the data from the previously mentioned BRF14 trial, the general recommendation is that imatinib should be reintroduced at the same dose as recommended for first line. Evidence II, B.

#### Treatment for patients with disease progression following imatinib failure

The first step to undertake when dealing with advanced GIST patients, who have progressed despite imatinib treatment, is to ensure treatment adherence and check for drug interactions that might decrease efficacy. Consideration may also be given to determine plasma imatinib concentrations, to better analyze these issues [78]. If there is proper treatment compliance, systemic treatment will have to be modified.

*Imatinib dose escalation.* The first recommended therapeutic maneuver consists of increasing the dose of imatinib to 800 mg/daily. The decision is based on results of crossover to 800 mg after disease progression on 400 mg in EORTC phase III trial studies [79] and American Intergroup (study S0033) [68]. In both cases, the observed progression-free rates in patients receiving a higher dosage were 30–35 %. The median time to progression was 3–4 months. However, in one of the studies, 18 % of patients remained progression free during 1 year. The incidence of anemia and asthenia increases significantly with this dosage; therefore, a strict follow-up is required. Results from a retrospective EORTC trial indicated that 800 mg dose is more effective than 400 mg in patients who have KIT exon 9 mutations [46]. These data were published recently in a meta-analysis including 722 patients [48].

*Sunitinib* is a multitargeted or selective TKI active inhibitor that is active against alpha-type and beta-type PDGFR and VEGFR receptors. Results of a randomized phase III trial versus placebo revealed a prolongation of the time to progression from 1.5 to 6.3 months in patients with GIST who progressed despite imatinib treatment [80]. Accordingly, it has been approved by the EMA and FDA as treatment for GIST resistant to imatinib therapy and for those who do not tolerate it. The recommended dose is 50 mg orally once a day during 4 weeks followed by a 2-week rest period, although an uninterrupted daily dose of 37, 5 mg is a valid alternative [81]. The most common side effects were asthenia, skin toxicity, diarrhea, hypertension and hypothyroidism. A prospective study showed an increased drug efficacy in patients with wild-type KIT GIST or mutations in exon 9 and 11 [49]. Likewise, patients who benefited most from sunitinib treatment were those with secondary KIT mutations in exon 13 and 14 compared to those with exon 17 and 18 mutations.

*Regorafenib* is a second generation TK inhibitor targeting KIT, RET, BRAF, VEGFR, PDGFR and FGFR. A median PFS of 4.8 months with imatinib, sunitinib and regorafenib versus 0.9 months with placebo was observed in a randomized trial for patients with refractory GIST. The most frequent side effects were high-blood pressure, hand-foot syndrome and diarrhea [82]. The only available preliminary data showed a correlation between genotype and regorafenib sensitivity, suggesting significant activity against KIT exon 11 mutations and those found in the KIT-activating mutations loops as well as some forms of KIT wild type. Regorafenib has the FDA approval and is currently undergoing assessment by the EMA as treatment for advanced GIST after failure of imatinib and sunitinib.

The available treatment options following imatinib, sunitinib and regorafenib administration (the latter following approval of regulatory authorities) are still in the experimental phase. The first recommendation is to offer these patients the opportunity to participate in clinical trials conducted under an investigational new drug. If this is not possible, an individual treatment can be scheduled for selected patients with other drugs such as:

*Nilotinib* is a second generation TK inhibitor active in chronic myeloid leukemia and with the inhibitory effect of KIT and PDGF. Preliminary results of a phase III trial comparing nilotinib with imatinib, sunitinib or supportive care in resistant GIST patients whose disease had progressed to imatinib and sunitinib did not show differences in PFS and OS treatment groups. On the other hand, significant differences in overall survival were noted when the analyses were limited to those who received nilotinib as strictly third-line treatment (excluding those who had received additional therapy) [83].

*Sorafenib* is a VEGFR, KIT, PDGR and BRAF inhibitor. Preliminary results of a phase II trial showed activity in imatinib-resistant and sunitinib-resistant patients with acceptable tolerance. This could be an alternative until regorafenib becomes available [84].

*Imatinib* and doxorubicin combination: Promising activity was been observed in a GEIS group study with doxorubicin dose of 20 mg/m^2^/once a week, which could be particularly suitable for patients with wild-type GIST [85].

### Response evaluation

An abdominal and pelvic CT with contrast and image acquisitions of the arterial and portal phases allows identification of hypervascular hepatic lesions that would otherwise go unnoticed and become evident when they become hypodense with treatment. Choi criteria [74] combine changes in both size (RECIST) and density measures (Hounsfield Units: HU). Responses can mimic progression due to the increasing size of some lesions that can only be interpreted if HU are considered [75]. On the contrary, a CT scan without endovenous contrast detects hemorrhage or intratumoral calcification.

Other techniques such as MRI are strictly limited to hepatic studies, complex locations such as the rectum [76] and allergic reactions to iodine contrast, since evaluation of HU is not feasible. PET is reserved for inconclusive cases by other techniques such as CT or MRI or the early assessment of response to imatinib [77]. However, PET is useful for early detection of responses, mandatory in some neo-adjuvant indications.

Both RECIST 1.1 and Choi [74] criteria must be taken into account to avoid confounding it with pseudoprogression due to myxoid degeneration or intratumoral hemorrhage (Table 5).

**Table 5:** Response evaluation criteria

	RECIST	PET	Choi criteria
Complete response (CR)	All lesions must disappear	Lack of FDG uptake	All lesions must disappear
		Unable to distinguish it from surrounding tissue	No new lesions
Partial response (PR)	Decreasing size 30 % of sum of target lesions	Decreasing size 15–25 % of SUV after 1 cycle and more than 25 % after subsequent cycles	Decreasing size >10 % or decreasing density ≥15 % HU
Stable disease (SD)	Between PR and PD	<25 % increase or SUV decreases <15 %	Does not fufil CR, PR or PD criteria
			No symptom deterioration due to tumor progression
Progressive disease (PD)	Target lesions increase >20 %	SUV increases >25 % or new lesion uptake	Size increases >10 % without density decreasing
			New intratumoral nodules
			Size or tissue part of hypodense lesion increases

*FDG* fluorodeoxyglucose, *PET* positron emission tomography, *SUV* standardized uptake volume, *HU* Hounsfield units

## Follow-up of patient diagnosed with gist

An abdomen and pelvic CT with contrast and image acquisitions of the arterial and portal phases is the method of choice for initial diagnosis and follow-up for GIST. Due to low metastatic frequency of pulmonary metastases (2 %) [86], thoracic imaging study is only indicated based on clinical suspicion. There are no studies analyzing the efficacy of different follow-up strategies. Follow-up will be stratified based on risk, size, number of mitosis and location according to the Miettinen classification [9].

Other techniques such as MRI are strictly limited to hepatic studies, complex locations such as the rectum and allergic reactions to iodine contrast given that evaluation of Hounsfield units is not feasible. PET is reserved for inconclusive cases by other techniques such as CT or MRI or the early assessment of response to imatinib [77].

### Localized resectable disease

Follow-up after resection according to risk group:

Very low risk: Routine follow-up is not indicated due to the low-risk of relapse- even if it is not null.

Low risk: Follow-up can be carried out every 6 months at diagnosis up to the fifth year.

Intermediate high risk: Patients receiving adjuvant imatinib treatment require regular monitoring with clinical analysis every 3 months to control drug tolerance. A follow-up CT scan should be done every 3–6 months during adjuvant treatment. The highest risk of recurrence takes place in the first 2 years. After this period, a CT scan should be performed every 3–6 months up to the fifth year and annually up to 10 years.

### Localized unresectable or metastatic disease

Follow-up should be conducted every 3 months since the beginning and can be prolonged up to every 6 months if response is obtained, especially if response remains beyond a 5-year period. Both RECIST 1.1 and Choi [74] criteria must be taken into account to avoid confounding it with pseudoprogression due to myxoid degeneration or intratumoral hemorrhage as previously described.

## Special cases

### Neoadjuvant and induction therapy

Systemic induction therapy has the aim of facilitating surgery through tumor shrinkage, whereas systemic neoadjuvant therapy targets survival advantage [87].

In locally advanced and unresectable GIST, there are few cases that would eventually become resectable after induction treatment with imatinib [88]. However, for those locally and resectable GIST for which a mutilating surgery is planned, a cytoreductive treatment with imatinib should be attempted. Thus, in gastric GIST to avoid subtotal gastrectomy, duodenal GIST near the ampulla of Vater or rectal GIST to avoid abdominoperineal amputation.

Early assessment of response is needed to minimize risks since a delay could further hamper surgery after an unsuccessful imatinib treatment. Mutational analysis should be mandatory due to the robust genotype predictive biomarker (IIb). Hence, exon 9 mutants would require 800 mg/day of imatinib; D842 V mutants no induction treatment would be active and for those KIT/PDGFRA wild type, is doubtful that imatinib could be active enough. A CT scan can be of some usefulness for assessing early response, but the use of PET scan after induction therapy seems more advantageous given its ability to verify the efficacy of the treatment within a very short time [89].

The recommended duration of preoperatory treatment cannot be based on objective criteria. However, it is estimated that surgery could be performed within 6–12 months after starting imatinib, since maximal response and minimal risk of secondary resistance is expected in this time interval.

#### Final recommendations

1. There is a lack of published evidence regarding neoadjuvant treatment in operable GIST and therefore should not be used outside clinical trials.
2. Induction treatment could be recommended in individual basis with the aim of offering a less mutilating/function sparing surgery.

### Small GIST<2 cm

Many small GIST, <2 cm, are incidental findings during surgeries carried out for a number of other reasons. A small GIST found accidentally in a surgical specimen does not require any additional therapeutic procedure.

In those uncommon cases of small GIST diagnosed before surgery, the excision is not clear enough and a shared decision-making process with the patient should be offered.

Anyway, the mitotic index of those tumors should be taken into account, although the incidence of small size and high mitotic index is very low according the literature.

### Focal progression

There is a well-documented type of secondary resistance to imatinib called nodule within mass. This is a focal disease progression, while most of the tumor burden is still under control. For these cases, maintenance of systemic treatment (generally imatinib) as well as applying local measures, can keep the patient free of progression for over a year in one-third of cases (xii). It is the treatment of choice over second-line systemic treatments.

The most common local treatments are surgery, radioablation and arterial embolization. In the absence of controlled trials, the choice of treatment should be based disease characteristics as well as on an experienced medical team.

### Final recommendations

- Systemic therapy should not be interrupted or replaced when progression is limited to a single or only a few focal points amenable to local treatment.
- Choice treatment for focal progression is maintenance of systemic therapy together with local control techniques appropriate for each case.
